# Metabolic diagnosis and medical prevention of calcium nephrolithiasis and its systemic manifestations: a consensus statement

**DOI:** 10.1007/s40620-016-0329-y

**Published:** 2016-07-25

**Authors:** Giovanni Gambaro, Emanuele Croppi, Fredric Coe, James Lingeman, Orson Moe, Elen Worcester, Noor Buchholz, David Bushinsky, Gary C. Curhan, Pietro Manuel Ferraro, Daniel Fuster, David S. Goldfarb, Ita Pfeferman Heilberg, Bernard Hess, John Lieske, Martino Marangella, Dawn Milliner, Glen M. Preminger, Jose’ Manuel Reis Santos, Khashayar Sakhaee, Kemal Sarica, Roswitha Siener, Pasquale Strazzullo, James C. Williams, The Consensus Conference Group, R. Bartoletti, N. Buchholz, D. Bushinsky, G. B. Capasso, E. Cicerello, F. Coe, E. Croppi, A. Cupisti, G. C. Curhan, J. Desai, A. Fabris, P. M. Ferraro, D. Fuster, G. Gambaro, D. S. Goldfarb, I. P. Heilberg, B. Hess, Ph. Jaeger, Z. Kirkali, D. Kok, E. Letavernier, J. Lieske, J. Lingeman, M. Marangella, S. Mazzaferro, O. Moe, D. Milliner, A. Nouvenne, G. M. Preminger, D. Prie, J. Reis Santos, D. Rendina, K. Sakhaee, K. Sarica, R. Siener, L. Soldati, P. Strazzullo, A. Tasca, A. Trinchieri, G. Vezzoli, C. Vitale, W. Wu, J. C. Williams, E. Worcester

**Affiliations:** 1 Department of Nephrology and Dialysis, A. Gemelli University Hospital, Catholic University of the Sacred Heart, Rome, Italy; 2 A.S.L. 10, Florence, Italy; 3 Department of Nephrology, University of Chicago Medicine, Chicago, USA; 4 Department of Urology, Indiana University School of Medicine, Indianapolis, USA; 5 Department of Internal Medicine, Southwestern Medical Center, University of Texas, Dallas, USA; 6 Department of Urological Surgery, Sobeh’s Vascular and Medical Center, Dubai, UAE; 7 Department of Nephrology, Medical Center, University of Rochester, Rochester, USA; 8 Renal Division, Brigham and Women’s Hospital, Boston, USA; 9 Department of Nephrology, University of Bern, Bern, Switzerland; 10 Department of Nephrology, New York Harbor VA Health Care System, New York, USA; 11 Department of Nephrology, Sao Paulo University, Sao Paulo, Brazil; 12 Department of Internal Medicine and Nephrology, Klinik Im Park Hospital, Zurich, Switzerland; 13 Department of Laboratory Medicine and Pathology, Mayo Clinic, Rochester, USA; 14 Department of Nephrology, A.S.O Ordine Mauriziano Hospital, Turin, Italy; 15 Department of Nephrology, Mayo Clinic, Rochester, USA; 16 Department of Urology, Duke University Medical Center, Durham, USA; 17 Department of Urology, Catholic University of Portugal, Lisbon, Portugal; 18 Southwestern Medical Center, Mineral Metabolism Research, University of Texas, Dallas, USA; 19 Department of Urology, Dr. Lutfi KIRDAR Kartal Research and Training Hospital, Istanbul, Turkey; 20 Department of Urology, University of Bonn, Bonn, Germany; 21 Department of Internal Medicine, University of Naples, Naples, Italy; 22 Department of Anatomy and Cell Biology, Indiana University School of Medicine, Indiana, USA

**Keywords:** Nephrolithiasis, CKD, Bone disease, Diet, Beverages, Renal tubular acidosis

## Abstract

**Background:**

Recently published guidelines on the medical management of renal stone disease did not address relevant topics in the field of idiopathic calcium nephrolithiasis, which are important also for clinical research.

**Design:**

A steering committee identified 27 questions, which were proposed to a faculty of 44 experts in nephrolithiasis and allied fields. A systematic review of the literature was conducted and 5216 potentially relevant articles were selected; from these, 407 articles were deemed to provide useful scientific information. The Faculty, divided into working groups, analysed the relevant literature. Preliminary statements developed by each group were exhaustively discussed in plenary sessions and approved.

**Results:**

Statements were developed to inform clinicians on the identification of secondary forms of calcium nephrolithiasis and systemic complications; on the definition of idiopathic calcium nephrolithiasis; on the use of urinary tests of crystallization and of surgical observations during stone treatment in the management of these patients; on the identification of patients warranting preventive measures; on the role of fluid and nutritional measures and of drugs to prevent recurrent episodes of stones; and finally, on the cooperation between the urologist and nephrologist in the renal stone patients.

**Conclusions:**

This document has addressed idiopathic calcium nephrolithiasis from the perspective of a disease that can associate with systemic disorders, emphasizing the interplay needed between urologists and nephrologists. It is complementary to the American Urological Association and European Association of Urology guidelines. Future areas for research are identified.

## Introduction

The present document is the result of a Consensus Conference held in Rome on March 26–28, 2015 that concluded the work of an International Faculty of experts in the field of renal stones. The faculty was multidisciplinary, representing nephrology, urology, nutrition, internal medicine, endocrinology, and laboratory medicine, which was desirable in view of the multifaceted nature of nephrolithiasis and in line with the scientific ‘tradition’ in this field.

In 2014 and 2015, three clinical practice guidelines addressing the medical management of nephrolithiasis were published: the American Urological Association (AUA) [1], American College of Physicians (ACP) [2], and European Association of Urology (EAU) [3] guidelines. These guidelines underlie the interest in this condition that is increasing in prevalence, and is difficult to treat because of its unpredictability, complexity and heterogeneity. Last but not least, they also highlight the need for increased knowledge regarding key pathogenic aspects and newer treatment strategies.

Why another consensus statement on nephrolithiasis? The answer is relatively easy, expressed as three goals:

1. To address questions that have not been addressed by previous documents;
2. To address those questions with a more general perspective for a disease that can be associated with systemic disorders.
3. To emphasize primarily the interplay between urologists and nephrologists, but also with other medical experts in the management of this disorder.

Of course, it is not all black and white, in the sense that some of the questions, or some of the relevant systemic disorders, were in part also addressed by other documents. However, in general, this statement is complementary to the other recent documents; in the areas of overlap, it partially endorses the AUA and EAU guidelines with a few modifications. This consensus statement was born in a strong mixed uro-nephrological collaboration with an accent on some typical renal medicine issues, i.e. chronic kidney disease (CKD), metabolic bone disease (MBD), and with an attempt to define the areas of cooperation.

Furthermore, different from other documents, some of the questions discussed in this statement represent the cutting edge of a relevant new understanding of the disorder (i.e. question #14: Use of surgical observations for diagnosis); and some are relevant not only from a good clinical practice perspective, but also for future research. For example, this statement addresses how to define the clinical phenotype of the idiopathic calcium nephrolithiasis (CN) patient, and the definition of clinical activity of the CN, both useful in designing randomized clinical trials (RCT), that are largely lacking in this field of medicine.

This document addresses only the so-called idiopathic CN; non-idiopathic calcium stones, uric acid, cystine and struvite stones are addressed just for those aspects needed to identify and manage the idiopathic CN.

## Methods

A Steering Committee which was set up by a previous conference (Nephrolithiasis: a systemic disorder, Rome, March 21–23, 2013) discussed which questions should be asked; these were selected because of clinical relevance, and/or the insufficient consensus on them, and/or not having been addressed by other documents, and/or to address topics for the development of cooperation between nephrologists and urologists. Twenty-seven questions were, in the end, proposed to a panel whose members were selected according to their expertise in nephrolithiasis and allied fields.

The task of the Steering Committee was to develop recommendations based on the analysis of the literature or on the consensus of the Faculty for best clinical practice in the management of CN patients. The whole Faculty, including the Steering Committee, comprised 44 members which were divided into working groups of 4–5 members each to analyse ahead of the conference the relevant literature, and to carry out, whenever necessary, on-line surveys among the community involved in the treatment of renal stone patients, including urologists and nephrologists and other specialists (clinical epidemiologists, nutritionists, biochemists, etc.). Working groups addressed 2–4 questions each. All the work and preliminary statements developed by each group were presented to the Faculty in plenary sessions in Rome where they were exhaustively discussed during 1.5 days, at the end of which each working group met to revise their statements in view of the plenary discussions and the results of other working groups’ possibly interrelated results. The following day, the revised versions of all statements were presented to the Faculty, discussed and approved. Following the conference, the consensus statement draft prepared by members of a writing committee was submitted to all members of the Consensus Conference Group for final revision.

### Analytical procedure

The groups performed a systematic review of the literature to retrieve all randomized clinical trials (RCT) that investigated topics relevant to their assigned questions. Cohort and case–control studies in addition to RCTs were also considered in the analysis due to the very small number of RCT-based evidence available in the literature.

Using appropriate search strategies, potentially relevant titles and abstracts published up to June 2014 were retrieved, from which 5216 potentially relevant articles were selected; from these, 3855 records were excluded based on title/abstract; 954 were excluded after screening the abstract or reading the article. In the end, 407 articles were deemed to provide useful scientific information.

The definition of the scientific ‘strength’ of each statement was based on the AUA categorization (http://www.auanet.org/education/guidelines/management-kidney-stones.cfm).

## Questions and consensus statements

### Questions #1 and #2

In a registry cohort study, one or more stone episodes were associated with an increased risk of ESRD [adjusted hazard ratio (HR) 2.16, 1.79–2.62], new stage 3b-5 CKD (1.74, 1.61–1.88), and doubling of serum creatinine (1.94, 1.56–2.43). However, the absolute increase in the rate of ESRD associated with stones was small (2.48 per million person days in people with stones versus 0.52 in people without) [4].The risk seems to be greater in women than in men [4, 5]. In the Olmsted County cohort studies, those stone formers who developed CKD or ESRD were more likely to have a history of hydronephrosis, struvite stones, recurrent UTI, acquired single kidney (15 vs. 3 %), neurogenic bladder (12 vs. 1 %), and ileal conduit (9 vs. 0 %) [6, 7]. Among the Third National Health and Nutrition Examination Survey (NHANES III) participants, a history of kidney stones in subjects with body mass index (BMI) ≥ 27 kg/m^2^ increased the probability of having an estimated glomerular filtration rate (eGFR) < 60 ml/min/1.73 m^2^ compared to overweight non-stone formers [8]. Stone formers with cystinuria, uric acid or struvite stones, renal tubular acidosis, or chronic bowel disorders frequently manifest decreased GFR, and CKD/ESRD [9, 10]. Standard Roux-en-Y gastric bypass and malabsorptive types of bariatric surgery are both associated with an increased risk for stones and CKD [11]. In a multicenter registry study on 5,745 patients undergoing percutaneous nephrolithotomy (PCNL), the risk of CKD was associated with the number of procedures for stone removal [12].

Low quality studies do not permit a confident conclusion on the relevance of kidney damage induced by urological stone procedures and the development and progression of CKD. The high-speed of technological advancements in urology does not easily allow for comparison between study cohorts, even when investigated over a short space of time. Although noninvasive and mini-invasive urological procedures for stone removal do damage the kidney to a certain extent, the differentiation of the renal damage due to the urological procedure versus the stone disease itself is a challenge.

*Future research directions*

- Studies to evaluate the risk of CKD according to etiology and composition of renal stones.
- Registry studies on the effect of different urological treatments of renal stones. This requires a minimum standard for data acquisition (e.g. stone burden, stone composition, concomitant obstruction, concomitant infection, urological procedure(s), repeated procedures.
- Methods for the evaluation of the renal damage in stone formers.

### Questions #3 and #4

Experts surmise that patients with hypercalciuria often excrete more calcium than they absorb, indicating a net loss of total body calcium. The source of this additional urine calcium is thought to originate from the skeleton. Hypercalciuric stone formers exhibit decreased bone mineral density (BMD) and the decrease is correlated with an increase in urine calcium excretion [13]. The decreased BMD also correlates with an increase in markers of bone turnover, as well as increased rates of fractures [14]. Patients with hypercalciuria, especially those who are at increased risk for osteoporosis, should have bone density determined by DXA. There are few controlled studies in hypercalciuric stone formers with low bone mass [15, 16]; however, there is a wealth of data in patients with osteoporosis to guide therapy. Patients should consume an age- and gender-appropriate amount of dietary calcium and should be 25-hydroxyvitamin D replete. Pharmacologic therapy that is directed at reducing recurrent stone formation may also help stabilize bone density. Thiazide diuretics lower urinary calcium and may increase bone density [17]. Alkali decreases bone resorption, especially in patients eating a high animal protein diet, and may improve bone mass as well [18, 19].

*Future research directions*

- Studies to evaluate which stone formers are at most risk of bone disease.
- Studies to evaluate which treatment(s) for recurrent stones most favorably increase bone mass.
- Studies to evaluate which treatment(s) for osteoporosis in stone formers most favorably increase bone mass.

### Question #5

To most, ‘idiopathic calcium nephrolithiasis’ means stones known or suspected to be made of calcium oxalate and/or hydroxyapatite without a systemic cause. Whether or not hypercalciuria is part of the definition is controversial. The relevance of this question is two-fold. First, diagnosing a stone patient as idiopathic means that a definitive treatment of a specific etiology, if available, cannot be offered to him; on the contrary, patients with idiopathic nephrolithiasis will be amenable only to a ‘generic’ pathophysiological treatment addressing life style, nutritional, and urinary risk factors. Second, how ‘idiopathic stones’ are defined is relevant to clinical research. In fact, studies may not be comparable if the definition of this prevalent condition differs between studies.

Idiopathic stone disease is a diagnosis of exclusion but individual practitioners’ lists of known causes to be ruled out are not the same. Table 1 lists the criteria specifically indicated in articles by some of the Faculty members [20–29]. Note that few articles in the list address the calcium stone former with ‘idiopathic hypercalciuria’ [20–25]. The majority address generic ‘idiopathic calcium stones’ where the calcium nature of the stone has been ascertained by stone analysis or implied by radio-opacity. On the contrary, the precise stone composition is not generally a part of the definition of the phenotype. However, according to the Coe and Lingeman [21], idiopathic calcium stone formers should be restricted only to those forming predominant calcium oxalate (CaOx) stones (with only a marginal amount of hydroxyapatite), while hydroxyapatite and brushite stone formers are distinct. The Faculty has agreed that hydroxyapatite stone formers and first-time stone formers with some brushite in the stone should be investigated for systemic causes as well.

**Table 1:** Calcium nephrolithiasis: conditions to be ruled out in the idiopathic form

1. Hyperparathyroidism
2. Hyperthyroidism
3. Sarcoidosis
4. Vitamin D excess
5. Calcium supplements
6. Prolonged immobilization
7. Clinical evidence of bone disease
8. Malignant neoplasms
9. dRTA
10. MSK
11. Primary hyperoxaluria
12. Enteric hyperoxaluria
13. Bowel disease
14. Chronic pancreatitis
15. Vitamin C supplements
16. Chronic diarrhea
17. Lithogenic drugs
18. Urinary infection
19. Gouty diathesis
20. Cystinuria

This list is derived from [20–29]

*dRTA* distal renal tubular acidosis, *MSK* medullary sponge kidney

The list could be different and more focused if the precise stone composition is known, but if this is not available the extended list of 20 items (see Table 1) should be used. Note that this list and the following to be applied in specific conditions (Tables 2, 3) are mere opinion-based guidelines.

**Table 2:** Conditions to be excluded in idiopathic CaOx nephrolithiasis with hypercalciuria, hypocitraturia and hyperoxaluria

Conditions	**Hypercalciuria**	**Hypocitraturia**	**Hyperoxaluria**
Primary hyperparathyroidism	∙		
Prolonged immobilization	∙	∙	
Incomplete dRTA	∙	∙	
Drugs and vitamin excess	∙ (vit. D)		∙ (orlistat, vit. C)
Chronic diarrhea		∙	
Chronic pancreatitis, Crohn’s disease, gastric bypass procedures, or small bowel resections		∙	∙
Nephrocalcinosis	∙	∙	∙
Genetic conditions associated with stones (including primary hyperoxaluria)	∙	∙	∙
MSK	∙	∙	

Abbreviations, see Table 1

In reference to ***idiopathic CaOx*** stone disease, it is worth noting that this name represents a heterogeneous group of diseases, because a given patient may have ‘idiopathic hypercalciuria’, ‘idiopathic hypocitraturia’ or ‘idiopathic hyperoxaluria’, or none of these urinary risk factors.

The Faculty considered that it is reasonable to exclude the conditions listed in Table II in a stone practice. Since it is not possible or advisable to perform systematic genetic studies due to the rarity of these conditions, they should be ruled out only in those cases where there is clinical suspicion [30].

In reference to ***hydroxyapatite stones***, hydroxyapatite is a frequent constituent of calcium stones. Its representation in a stone in a non-marginal quantity could suggest secondary forms of nephrolithiasis. The following conditions should be ruled out before concluding that a hydroxyapatite stone former is idiopathic: primary hyperparathyroidism, renal phosphate wasting conditions, medullary sponge kidney (MSK), complete and incomplete distal renal tubular acidosis (RTA) (see below, questions #6 and #7), abuse of absorbable antacids (calcium carbonate, the ‘modern’ form of *milk alkali syndrome*) and drugs inducing proximal RTA (carbonic anhydrase inhibitors).

In reference to ***brushite stones***, their clinical phenotype has probably changed in recent decades. In case series recently described, the profile of the brushite stone former is one of a recurrent stone patient who had multiple previous extracorporeal shock wave lithotripsies (ESWL), and who may have converted from another stone composition such as calcium oxalate [31, 32]. Hypercalciuria is almost invariably present, but complete distal RTA and primary hyperparathyroidism are not the cause [32]. The finding of obstructive lesions of the urinary tract, either congenital or acquired, is particularly notable in these stone patients [33]. The clinical phenotype is probably different in first-time stone formers with a brushite composition. Actually, in the older case series described by Pak et al.[34] distal RTA was frequently observed in brushite stone formers. Series suggest that idiopathic recurrent CaOx stone formers may switch to hydroxyapatite or brushite stones over time [35]. This is hypothesized to be the consequence of distal nephron damage induced by ESWL or previous obstructive episodes, or of preventive treatment with alkaline citrate [33–36]. In these hydroxyapatite or brushite patients, no investigation for systemic condition is warranted. On the contrary, in those patients who have passed brushite or apatite stones for the first time, systemic causes should be looked for.

The Faculty notes that the definitions used in this section are based on 24-h urine collection, although circadian variations in urine composition could be of great interest and value in the pathogenesis of idiopathic calcium stone disease.

*Future research directions*

- Studies on the pathogenesis of hypercalciuria
- Studies on the pathogenesis of brushite stones
- Studies on the relationship between ESWL and brushite stones
- Studies on circadian variation in urine composition
- Discovery of urinary markers of idiopathic calcium nephrolithiasis and metabolic activity

### Questions #6 and #7

The incomplete distal RTA is a condition where urinary acidification is defective but, at odds with the complete form, there is no systemic acidosis. Most incomplete distal RTA cases are not diagnosed in common practice because they are not even suspected or because of the complexity of the diagnosis that requires the classical short NH_4_Cl loading test. Incomplete distal RTA can be an acquired condition (e.g. nephrocalcinosis, MSK, Sjögren’s syndrome, obstruction, repeated ESWL), but it is conceivable that incomplete distal RTA is in part due to allelic variants of genes recognized to cause the overt form. Indeed, in a family carrying an autosomal-recessive V-ATPase B1 subunit mutation, some heterozygous members were also affected by recurrent CN [37].

Different test types and durations, and varying cut offs for urinary pH for the definition of incomplete distal RTA have been proposed. However, due to lack of rigorous comparative studies, the validity of the different provocative test protocols in patients with (and without) recurrent CN is currently unknown. With this limitation, incomplete distal RTA prevalence seems to be present in between 2 and 21 % of the general calcium stone forming population [38, 39]. Thus, incomplete distal RTA is a relatively frequent condition in recurrent CN. As the stone composition changes from CaOx to mixed CaOx-hydroxyapatite to pure hydroxyapatite, the prevalence of incomplete distal RTA increases from 5 to 40 % [40]. Thus, incomplete distal RTA should be suspected in unexplained recurrent nephrolithiasis when stones are composed mostly or exclusively of hydroxyapatite; it should also be considered in recurrent calcium stone formers (either CaOx or hydroxyapatite) with hypocitraturia and less acidic urinary pH and in patients with nephrocalcinosis. Although no published data on 24-h urinary pH or fasting urinary pH in patients with incomplete distal RTA is available, the Faculty has agreed that in fasting urine a pH > 5.8 should suggest the possible existence of an incomplete distal RTA.

There are no RCTs for the prevention of stones in patients with incomplete distal RTA. In small studies, treatment with alkali citrate in adults with incomplete distal RTA decreased hypercalciuria, increased citraturia and reduced stone recurrence [41, 42].

*Future research directions*

- Clinical and basic studies in the area of incomplete distal RTA and in CN associated with incomplete distal RTA.
- Studies in which 24-h or fasting urinary pH should be used as a guide to indicate which stone formers should be tested for incomplete distal RTA.
- Studies on easier test protocols for diagnosing incomplete distal RTA.

### Question #8

**Table 3:** Conditions to be excluded in hyperuricosuric calcium urolithiasis

Overproduction	Renal leak
Gout/metabolic syndrome	Uricosuric agents
Dietary purine overload	Rare transporter diseases
Catabolic states	
Rare monogenic diseases of purine metabolism	

The coexistence of CaOx and uric acid in urolithiasis has been noted repeatedly since the 1890s; Prien and Prien observed that gouty patients passed stones which contained or were composed of calcium oxalate; the first proposal that HUCU represents a novel syndrome was offered by Coe and Raisen in 1973 [43–45].

Since urine calcium and urine uric acid are correlated it should not be surprising that there exists a group of patients who have ‘high’ levels of both. Actually, epidemiologic studies of a large population of uncharacterized stone-formers failed to identify an independent association between urinary uric acid excretion rate and risk of all kidney stones [46]. On the contrary, earlier studies overlooked the fact that calciuria and uricosuria are not independently associated.

Indication of HUCU as a distinct entity mainly derives from the ‘disputed’ pathophysiological links between uric acid and CaOx crystallization [47–50]. Actually, several models have been proposed that are not mutually exclusive and can easily coexist. In fact, they can act in concert to cause CaOx crystallization. The models include the salting out of CaOx by urate in solution, the sequestration of various inhibitors of CaOx crystallization by colloidal uric acid, and heterogeneous nucleation or epitaxy of CaOx by uric acid/urate crystals [50–55]. Results from trials with allopurinol also support the idea of HUCU as a distinct entity; in these studies the risk of calcium stones using hard clinical outcomes was lowered [44, 56–59]. However, it remains to be demonstrated that the favorable effect goes via a urinary urate lowering effect. In view of the inconsistency of the definition of HUCU in older studies, the Faculty suggests a stricter definition of HUCU, i.e. mixed CaOx and urate stones in the presence of hyperuricosuria with or without other risk factors for calcium stones. This definition of HUCU permits inclusion of risk factors such as hypercalciuria, hypocitraturia, and hyperoxaluria but with hyperuricosuria acting as a major driver of lithogenicity. Hyperuricosuria in these patients is idiopathic and other diagnoses leading to hyperuricosuria should be ruled out (Table III). The Faculty notes that most of the clinical evidence and therapeutic trials did not address HUCU as here defined, particularly in reference to stone composition. Thus, there is need to revisit this condition in view of the proposed definition.

*Future research directions*

- Studies on the exact physicochemical basis and on the relative contributions of each model.
- Development of the in silico way (akin to EQUIL or JESS) of quantitatively predicting the risk of calcium stones conferred by urine uric acid/urate.
- Studies on dose–effect of uricosuria on calcium stone risk.
- Studies on the degree of lowering uricosuria that should be targeted.
- Studies on the interaction of hyperuricosuria with other urinary stone risks.
- Studies on whether the threshold to treat and the therapeutic target differ depending on the presence and degree of hypercalciuria, hypocitraturia, and hyperoxaluria.
- Studies on the mechanism of the allopurinol stone-preventive effect.
- Therapeutic trial on HUCU as here defined.

### Question #9

 All patients should have the composition of their urinary stones analyzed at least once. This is a cheap and valuable test that quickly identifies specific potential causes of stone disease. Importantly, a stone analysis alone can detect three important systemic causes of stone disease: cystine stones are diagnostic of cystinuria, dihydroxyadenine stones of APRT deficiency, and struvite stones of infection from a urease-positive organism. Other rare causes like drugs will also become apparent on a stone analysis.

A standard urinalysis with microscopy is an important screen for several systemic causes of stones. Pyuria suggests possible urinary tract infection. A high pH (>7.5) makes infection with a urease-positive organism possible, while a secondarily infected stone can be seen across the entire pH range. Proteinuria, especially in a male patient, makes it important to rule out Dent disease by confirming the nature of the proteinuria (i.e. screen for low molecular weight proteins like retinol binding protein, alpha-1 microglobulin, or beta-2 microglobulin). Crystals of cystine, dihydroxyadenine, and struvite are also characteristic in appearance, and these diagnoses can be confirmed with other tests.

A careful history is an essential part of a urinary stone evaluation. A history of any form of gastrointestinal disease suggests possible specific risk factors. Diseases that affect the small intestine or pancreas, including Crohn’s disease, gastric bypass procedures, or chronic pancreatitis, often lead to fat malabsorption and enteric hyperoxaluria. Gastrointestinal diseases that are associated with chronic diarrhea also cause excessive loss of bicarbonate in the stool and hence an appropriately low urinary pH, which raises the risk of uric acid stones. An intact colon is necessary for enteric hyperoxaluria. Hence, conditions with large gastrointestinal losses of fluid without a colon, e.g. the presence of an ileostomy, are typically associated with uric acid and not CaOx stones. Finally, certain fairly rare disorders such as sarcoidosis, or prolonged immobilization are associated with rapid loss of bone calcium and hypercalciuria.

A serum test for creatinine, calcium, phosphorus, K, Mg, HCO_3_, Cl, uric acid can effectively screen for important risk factors that influence stone risk factors and/or treatment choices. Hypercalcemia is an important sign of hyperparathyroidism, as well as rare conditions such as *CYP24A1* mutations or sarcoidosis. A low serum phosphorus is also consistent with hyperparathyroidism. A low potassium can contribute to hypocitraturia and/or suggest RTA, while a low serum bicarbonate suggests chronic gastrointestinal losses or RTA. An elevated serum creatinine suggests CKD, which can influence the choice of treatments like potassium citrate.

Systemic causes of urinary stone disease are associated with predictable urinary abnormalities. Hence, a 24-h urine for creatinine, pH, calcium, oxalate, citrate, uric acid, and qualitative cystine is useful to detect major causes of stone disease. For example, a low urine pH and low urine citrate suggests gastrointestinal base losses, and a high urine pH and low citrate indicates possible RTA. Hyperoxaluria makes it important to consider enteric or primary hyperoxaluria, and hypercalciuria makes it essential to consider a systemic disease of calcium metabolism. Conversely, if all elements of the 24-h urine are normal, a systemic cause of stone disease is less likely.

*Future research directions*

- Studies on the performance of different diagnostic algorithms in identifying secondary forms of nephrolithiasis.

### Question #10

Relative supersaturation (RS) of urine for calcium containing stone-forming species is the essential condition for a stone to form. RS can be easily estimated with a dedicated algorithm (Equil, JESS, Lithorisk). However, RS does not fully represent the overall lithogenic risk since it fails to estimate the inhibitory potential of urine and of the cells-crystal interaction in the renal tubule. There are no available data from pharmacologic RCTs with follow-up and treatment guided by urine supersaturation levels; hence, it is not established whether changes in urine supersaturation measurements predict reduced risk of recurrent stones with drug treatment. However, experts believe that measuring RS levels during follow-up may be a useful tool for increasing patient compliance.

The ULM is defined as the value of RS where spontaneous crystallization in urine occurs. It is different from RS because it is influenced by the inhibitory potential of urine. However, it still does not estimate the contribution of the cells-crystal interaction. Furthermore, ULM determination is time-consuming and not standardized.

*Future research directions*

- Studies on standardization of methods for ULM determination and application to larger numbers of stone forming and non-stone forming subjects.
- Studies investigating whether follow-up and treatment guided by urine RS determination decreases stone recurrences.

### Question #11

A major limitation of studies of crystalluria is the lack of standardization since urine can be examined fresh or stored, collected after fasting or as a 24-h study, centrifuged or evaporated, and quantified by coulter counter or microscope.

Studies in patients with CN have addressed frequency, composition, size and volume, agglomeration and attachment of crystals. Using fresh voided urine, it was found that crystalluria was more prevalent in calcium stone formers than in controls, but the type of crystals did not differ [60]. The type of CaOx crystals (whewellite vs. weddellite) seems to discriminate to a degree between hyperoxaluric and hypercalciuric stone formers [61]. Volume of crystals was higher in stone formers [62, 63]. However, crystal presence was not modified by different treatments even though the type of crystals changed from more calcium phosphate to more CaOx [60]. Presence of crystals was poorly related to degree of supersaturation [63].

Crystal agglomeration was more frequent in stone formers but of low incidence (1.4–8 %).

Inhibition of stone agglomeration exhibited some correlation with efficacy of treatment [64].

*Future research directions*

- Crystal aggregation (agglomeration) and crystal-cell interaction may be of clinical use in the future. At present, data are too scarce. For a better understanding of the mechanism of stone formation, it may be worthwhile to combine urine crystal analysis with endoscopic analysis of papilla, correlating it with the presence of plaques and plugs.

### Question #12

This question is flawed by the lack of a clear demonstration of a role of crystallization inhibitors and crystal aggregation inhibitors in CN. Furthermore, clinical value could only be obtained if:

1. it is known which inhibitors that affect the relevant processes differ between people who do vs. do not form stones,
2. measuring this difference can be done in a high throughput manner, and
3. the inhibitory action can be manipulated.

Studies on crystallization inhibition/inhibitors are numerous. However, they often lack standardization or were not designed to exactly mimic the environment where stones are thought to form by the two mechanisms described above. Study quality is low to moderate. The majority of studies dealt with individual urine compounds. Only a few studies include comparison of a stone former group to a control group. Even fewer studies actually compare inhibitory action of urine between the two groups. No studies were found that apply conditions resembling the renal interstitial fluid.

With respect to crystal growth, the results of the comparison between people who do or do not form stones are not uniform and only suggest the following expert opinion: urine contains many compounds that can inhibit crystal growth. In urine, macromolecules with an affinity for calcium deliver most of the growth inhibition capacity. However, when these compounds are lacking in quantity or function their action is taken over by other inhibitors that are also present in the urine. This overload of growth inhibitors may explain why crystal growth inhibition does not differentiate stone formers from controls.

With respect to crystal aggregation (or agglomeration) fewer studies are available but the conclusion is more uniform [65, 66]. Stone formers produce larger particles, mainly aggregates, and their urine is less able to inhibit crystal aggregation. Some studies show a correlation between the severity of the disease, expressed as number of stones formed per patient, and capacity to inhibit aggregation. This capacity seems to be exerted both by an interplay between macromolecules and small molecules like citrate [66]. Interventions that aim to increase the urine citrate content also increase the aggregation inhibition. There is no standardized method to measure crystal aggregation inhibition by whole urine in a high throughput method.

*Future research directions*

- Studies on the role of crystallization inhibitors and crystal aggregation inhibitors in stone formation in the interstitium (Randall’s plaque precursors), and in the interface with papillary deposits (Randall’s plaque), and ductal plugs.

### Question #13

Recent studies that have utilized endoscopy and papillary biopsy for the study of stone patients have revealed that stone formers differ not only in the types of mineral that they deposit in their stones (CaOx, brushite, struvite, etc.) but also in the pathology of their renal papillae with regard to the presence of Randall’s plaque and ductal plugging [26, 27, 33, 67–74]. Moreover, there is at least some correlation between the histopathologic pathologies observed in a papillary biopsy and the visual appearance of the same papilla before biopsy [75]. Thus, although stone formers have classically been divided by the minerals contained in the stones that they form [76], the possibility exists that this classification could be improved—or perhaps even replaced—by endoscopic observations during minimally invasive removal of renal stones.

The consensus of the working group was that endoscopic evaluation of papillae in stone formers could definitely have value, and that a scoring system for papillary pathology in stone formers has potential utility in diagnosis and prediction of prognosis of disease. We note that the first publications of a papillary scoring system have recently emerged [77–79].

Study of the utility of a papillary scoring system in stone formers calls for correlation with prognosis of disease and prediction of recurrence of stones. There is already some evidence for the correlation of papillary computed tomography (CT) density (which is increased by the presence of small stones or ductal plugs [80]) with stone recurrence rates [81]. Endoscopic observation of papillary pathology is more informative than CT [80], and thus logically could be more predictive of stone recurrence. Similarly, evidence for the connection of stones with CKD [82, 83] may well be informed by papillary pathology, as the plugging of collecting ducts is a probable cause of kidney injury. Finally, the working group was convinced that evaluation of papillary pathology endoscopically also could have the potential to indicate the direction of metabolic diagnosis and treatment for a given patient [84].

*Future research directions*

- Development of papillary scoring systems, including validation by external observers, with subsequent study of the utility of each scoring system in clinical practice.
- Studies of the correlation of papillary pathology with prognosis of disease and prediction of recurrence of stones.

### Question #14

Stone analysis is not uniformly well done around the world - or even uniformly well done within countries [85, 86]. Thus it is not surprising that data on stone composition have not been correlated well with patient outcomes. This is especially true for stone analysis done by wet chemical methods, where the errors inherent within such analyses [85, 87] would certainly preclude any ability to identify relationships between stone components and patient characteristics.

But even with the use of modern spectroscopic methods of analysing stone mineral, the existing practice has limitations. For example, evaluation of the composition of mixed stones relies on skilful dissection of the specimen, which may not always happen [86]. There are also several reports that suggest that the recognition of special stone morphologies can be pathognomonic for certain conditions [88–90], but there is no evidence that these morphologies are being widely recognized in analysis practice.

Although progress is being made in the use of dual-energy CT to identify stone composition [91], it is unlikely that this will ever replace stone analysis, as the spatial resolution required to assess stones of multiple components is quite fine [92]. Thus, the working panel concluded that work needs to be done to improve existing practices of stone analysis. Even though a detailed model for proper stone analysis exists, this model needs to be emulated by other sites. There is much need for education, as methods across countries are not consistent, and there are few practices for testing that are widespread [93]. In addition, the nomenclature for stone components needs to be standardized [86].

The working panel was convinced that stone analysis should be done, and because stone composition can change in a patient over time [94, 95], it should be done regularly and in a standardized fashion. In addition, although rare stones are indeed rare, stone analysis is inexpensive and delay in recognizing a rare stone in a patient can lead to loss of kidney function that would have been preventable if the rare stone had been recognized upon first presentation [96]. Although the panel was convinced that observation of stone morphology has potential utility, it concluded that more work needs to be done before this can be added to standard stone analysis.

*Future research directions*

- Educational work needs to be done to improve existing practices of stone analysis.
- Nomenclature for stone components needs to be standardized.
- More work is needed on stone morphology to implement it in stone analysis.

### Questions #15, #16 and #17

The distinction between single and recurrent stone formation is usually based on clinical evidence of either isolated or multiple episodes of stones, but this definition is not precise. Imaging studies complement the definition of stone recurrence. Stone recurrence and/or growth are indications of ‘metabolic activity’. The concept of metabolic activity of stone disease means the existence of an ongoing crystallization-driving process that most likely will lead to new stones or to the growth of already existing stones. It is the concept of metabolic activity that should ideally drive the clinician’s decision on how to treat the stone patient. Single and recurrent stone formers share many similarities in their metabolic profiles [97, 98]. There are no markers that may distinguish between single and recurrent stone formers and metabolically active vs. metabolically inactive stone formers. A nomogram (ROKS) for the prediction of a second (recurrent) symptomatic stone episode has been recently proposed [99]. However, only symptomatic stones were considered and the 10 years recurrence rate was only 30 % on the whole, and no more than 56 % in the subgroup of patients at highest risk. The evaluation of the stone patient should also address the patient’s risk factors for recurrent stone formation or other morbidities and indications. These risk factors and conditions include, but are not limited to, family history of stone formation; pediatric age; solitary kidney; patients with concurrent medical conditions; nephrocalcinosis, CKD or skeletal diseases; history of bowel disease or bowel surgery; patient’s job (i.e. pilots, frequent business travelers); difficult to treat stones (e.g. urinary tract abnormalities/reconstruction); and immuno-compromised conditions.

*Future research directions*

- Studies on markers that predict stone recurrence.
- Studies on metabolic markers that distinguish metabolically active vs. metabolically inactive stone formers.

### Questions #18 and #19

Follow-up is the mainstay of conservative and active management of CN, to prevent both stone growth and new stone formation. The greatest challenge in following stone formers is their low adherence to conservative or selective medical recommendations, particularly dietary advice. The purpose of follow-up is to assess the efficacy of treatment, to encourage patient compliance and ultimately reduce the risk of stone recurrence. Through close monitoring, the clinician is able to assess treatment adherence to or effectiveness of recommendations, allowing adjustment of pharmacological treatment dosing and the determination of both short- and long-term adverse effects of directed medical therapy. However, continued management of patients with stone disease varies widely between practitioners, since no studies have assessed an optimal schedule as the primary outcome. Despite the lack of evidence-based principles, systematic reviews of the literature and recently published guidelines help to establish which laboratory or imaging studies should be performed, their frequency and duration, and also to define the target population: single and/or recurrent stone formers [1, 2].

*Future research directions*

- Studies on the fate of asymptomatic stone formers. Should patients with renal stone(s) incidentally found in imaging studies undergo a metabolic evaluation and/or follow-up?
- New stone formation and stone growth are not always distinguishable depending on different imaging modalities. Future observational studies or RCTs should employ sensitive and, more importantly, standardized detection methods. They should define an absolute stone size or growth rate threshold(s) in order to ascertain the presence of baseline residual stones, and then to establish measurement of stone growth. The use of standard modalities and appropriate testing frequencies, to ascertain incident radiographic stones should be defined. Patients with asymptomatic stone growth, radiographic stone recurrence and/or asymptomatic stone passage should be followed untreated for several years for symptomatic stone recurrence to help determine whether and under what circumstances these measures can serve as appropriate surrogates for symptomatic stone recurrence. RCTs should separately report the outcomes of symptomatic and radiographic stones, describe the laboratory and radiographic testing that participants undergo, including their cumulative radiation exposure.

### Question #20

A low urine volume is one of the most important risk factors for urinary stone formation. One RCT showed that idiopathic CaOx stone formers who increased fluid intake to maintain a urine volume of greater than 2 l/day had a significantly lower recurrence rate during the 5-year follow-up period than the control group (12.1 vs. 27.0 %, p = 0.008) [100]. Another trial in CaOx stone formers who became stone free after shock wave lithotripsy found a decrease in stone recurrence in patients with increased fluid intake to achieve more than 2.5 l of urine per day compared with no treatment for 2–3 years (8.3 vs. 55.6 %, p < 0.05) [101].

By contrast, there is unfortunately no hard data on the importance of water composition. In both healthy subjects and patients with recurrent CaOx urolithiasis, an elevated intake of mineral water with a very high bicarbonate and magnesium content (28 and 44 mEq/l, respectively) was shown to produce higher urinary citrate and magnesium excretion compared to an equal amount of control water and to significantly alkalinize urine [102, 103]: nevertheless, the observed decrease in urinary super-saturation for CaOx was comparable to that obtained with the control water and mostly due to the increase in urine volume, whereas super-saturation for calcium phosphate was significantly increased due to the significant rise in urinary pH.

Most controlled clinical trials testing the effect of water with a high calcium content in calcium stone patients observed increases in urinary calcium excretion paralleled by a decrease in urinary oxalate excretion, presumably due to increased oxalate complexation by calcium in the gut and subsequent reduced intestinal absorption of free oxalate. However, the long-term effect on stone recurrence rate is unknown [104].

A prospective observational study on three large cohorts of individuals without a history of nephrolithiasis found that the risk of incident kidney stones was directly associated with consumption of sugar-sweetened beverages and inverse associations with the intake of orange juice but also with the consumption of both caffeinated and decaffeinated coffee, tea, beer and wine [105]. In a large RCT in stone-forming men with a high baseline sugar-sweetened beverage consumption, abstinence from soft drink intake significantly reduced the risk of recurrence at 3 years compared to the control group (33.7 vs. 40.6 %, p = 0.023) [106].

Overall, there is sound evidence against the consumption of sugar-sweetened beverages and circumstantial evidence in favour of orange juice intake with no added sugar for prevention of CN.

*Future research directions*

- Controlled trials of the effects of different types of beverages, including bicarbonate-rich mineral water and water with different calcium content, on the rate of stone recurrence in patients with CN are warranted.

### Question #21

An observational study supports the idea that the Dietary Approaches to Stop Hypertension (DASH) diet, an example of dietary model designed for the prevention of cardiovascular diseases, may be effective also in primary prevention of CN [107]. The Mediterranean diet [108] and the lacto-ovo vegetarian models [109] are other examples of healthy well-balanced diets with many features potentially effective for the prevention of CN.

It has been recognized that dietary recommendations tailored to the specific patient condition are more likely to be effective than non-specific dietary advice in preventing recurrences [110].

An RCT showed that an interventional diet consisting of normal calcium, low sodium and low animal protein content, associated with high fluid intake, effectively reduced the rate of stone recurrence as compared to high fluid intake associated with a low calcium diet [20]. In addition, high quality prospective studies showed that a normal dietary calcium intake (1000-1200 mg/day) for most adult individuals is associated with a reduced risk of stone formation compared to lower intakes [111–116]. Both clinical [117] and epidemiological studies [118] suggest to reduce sodium intake since the higher the salt intake, the greater the calcium excretion [119].

Large high quality cohort studies in the general population have demonstrated an inverse relationship between dietary calcium intake and urinary oxalate excretion [111, 120, 121]. When the recommended intake of calcium was achieved, even a relatively high dietary oxalate intake was not associated with higher CaOx stone risk [120]. Although these studies did not target recurrent CN patients, it is conceivable that patients with hyperoxaluria and a history of CaOx stones would also benefit from a relatively high calcium intake (1000-1200 mg/day) on the same grounds. As the urinary oxalate excretion is significantly related to the amount of oxalate in the diet^120^, a limited consumption of selected oxalate-rich foods is advisable in these patients. On the other hand, extreme restriction of dietary oxalate is impractical and possibly dangerous as it would result in lower intakes of fruits, vegetables and whole grains which are known to provide other major health benefits [120, 121].

A fruit and vegetable rich diet significantly increases the urinary citrate and phytate excretion [122] and was found to be protective against CN in a number of good quality observational prospective studies [20, 107, 123]. By contrast, a high dietary acid load reduces urinary citrate [124] and a good quality observational prospective study has shown that fish and poultry proteins are associated with increased risk of first-stone formation as opposed to vegetable and dairy proteins [125]. Thus, patients with hypocitraturia should be instructed to reduce non-dairy animal protein intake.

The results of good quality population-based prospective studies are consistent with a higher risk of CN in males taking vitamin C supplements [126, 127]. There is also circumstantial evidence in post-menopausal women that combined intake of vitamin D and calcium supplements increases the rate of incident CN [128].

*Future research directions*

- RCTs on the value of complex dietary patterns, such as the Mediterranean diet, in primary and secondary prevention of nephrolithiasis are warranted.

### Questions #22, #23 and #24

The relative merits and drawbacks of citrate and thiazides in stone prevention are well known but they have not been compared head-to-head in any trial. Discussion of the risks and benefits of both treatments should ensue with patients, whose values should be taken into consideration.

Citrate inhibits crystallization processes in calcium stone formers. Potassium citrate is therefore indicated for the following clinical situations: (1) recurrent CaOx and calcium phosphate stones with low or relatively low urinary citrate excretion; (2) patients with complete or incomplete distal RTA, chronic diarrheal states, drug-induced or diet-induced hypocitraturia [41, 42, 129–131], and known osteopenia or osteoporosis. In case of intolerance to citrate, potassium bicarbonate can be used instead. Sodium citrate should be avoided because sodium promotes calcium excretion and is less effective at promoting citrate excretion than potassium salts [132].

Thiazide diuretics should be offered to patients with high or relatively high urine calcium and recurrent calcium stones [133–138], although the precise mechanism of thiazide action has not been elucidated. The AUA guidelines [1] also suggest that thiazides would be useful in patients without relatively high urine calcium excretion as lowering urine calcium excretion may be effective regardless of the absolute rate of calcium excretion. Some studies showing efficacy of thiazides did not require hypercalciuria at baseline. Thiazides would be appropriate when dietary measures and increased fluid intake have not prevented stone recurrence. Under these circumstances, thiazides are considered appropriate for both CaOx and calcium phosphate stones. Appropriate doses and regimens include hydrochlorothiazide 25 mg twice a day, chlorthalidone 25 mg once a day or indapamide 2.5 mg once a day. Thiazides should always be administered with potassium to avoid increased cardiovascular risk and minimize glucose intolerance [139, 140]. Potassium citrate supplementation may be preferred to potassium chloride to maintain urinary citrate excretion, improve bone mineral density and reduce urinary calcium excretion. Alternatively, potassium loss can be counteracted by amiloride 5 mg or spironolactone 25 or 50 mg. Triamterene should be avoided.

Both citrate and thiazides appear to increase bone mineral density, a frequent correlate of hypercalciuria and calcium stones. Possibly these effects are synergistic though the combination has not been specifically tested. Thiazides may be considered the blood pressure lowering drugs of choice in patients with kidney stones as they have consistently been shown to be the preferred agents for the treatment of essential hypertension, systolic hypertension, and hypertension in the elderly because of their consistent effect to lower cardiovascular morbidity and mortality.

Treatment with oral phosphate results in reduction in urinary calcium excretion largely due to inhibition of calcitriol synthesis [141]. One main concern with oral phosphate administration has been the possibility of metastatic soft tissue calcifications resulting in renal functional impairment. One long-term, blinded controlled study in a small number of absorptive hypercalciuric kidney stone-forming subjects using sustained released potassium phosphate showed the safety of this preparation [142]. However, the efficacy was not tested in a multicenter trial using incident kidney stone episode as an outcome.

*Future research directions*

- RCTs on the pharmacological prevention with thiazides of recurrent calcium stone disease with different biochemical phenotypes (normo- and hypercalciuric, etc.).
- Since the pharmacological armamentarium for renal stone prevention is quite limited there is need of studies investigating new drugs to prevent stone recurrence.

### Question #25

In two open trials in patients with distal RTA and MSK prone to calcium phosphate stone formation, citrate treatment significantly reduced the prevalence of kidney stones [41, 42]. However, in one retrospective study it was shown that those who transformed the stone composition to calcium phosphate stones had a higher baseline and post treatment urinary pH compared to non-transformers [143]. Due to the sparsity of controlled studies delineating the risk of calcium phosphate stone formation with alkali therapy, alternatives to alkali treatment should be tested in prospective clinical studies. Until such a study is performed, clinicians should offer citrate treatment to patients with calcium phosphate stones in whom urinary citrate increases commensurate with a rise of urinary pH.

*Future research directions*

- Due to the potential risk of calcium phosphate stone formation with alkali treatment, alternatives to alkali treatment should be tested in prospective clinical studies using citric acid vs. potassium citrate or thiazide + potassium chloride vs. thiazide + potassium citrate in this population.

### Questions #26 and #27

 Nephrologists and urologists have been working side by side for a very long time. Yet, too often, there seems to be a rivalry, almost a competition, as can be found in many places between physicians and surgeons. Naturally, given the different approaches to disease, their view of the world and even their ‘dialects’ differ [144]. This poses a barrier between the two specialties that applies also to stone disease, which is often complex and caused by certain underlying co-morbidities that cannot be tackled by blasting the stone alone. Conveniently, though, for the urologists, developments in stone blasting technologies have made treatment very smooth and easy, leading to a neglect in looking for the diagnosis of underlying causes. On the other hand, nephrologists show only a limited interest in stone disease amongst all the other renal pathologies they have to deal with.

We believe that in the clinical management of patients with nephrolithiasis, the collaboration between nephrologists and urologists is crucial. A close collaboration based on each other’s understanding is needed not only during the acute phase of stone-related renal injury, but also during the clinically stable phase of the disease. However, in a global perspective this principle is not generally applicable and it needs to be emphasized that such a shift in routine cannot be accomplished without a change in the education of nephrologists. In a survey of urologists performed before the conference to obtain a better picture of their urologists’ point of view, 51 % of respondents claimed to refer stone formers to nephrologists “occasionally” and, more worrisome, 39 % said “never”. Yet, secondary and metabolic stone disease were the main reasons for referral to the nephrologist.

Currently, we are not aware of any guidelines for urologists regarding nephrology referrals. A recent study shows a poor implementation of guidelines among healthcare providers [145], and we may assume this to be true for urologists and nephrologists as well. Searches in literature databases for such guidelines using an array of search terms did not reveal any hits.

*Future research directions*

- As part of an integrated clinical management pathway for nephrolithiasis patients, the nephrologist and urologist—ideally in collaboration—will have to face the development of shared protocols for the evaluation of stone formers and for defining cooperation.
- The evaluation of *Quality and cost/benefit ratio* of the protocols for renal stone forming patients.
- *Cost/benefit analysis* of instrumental investigations for the follow-up of renal stone forming patients (economic cost, radiological risks).
